# Structural prediction of two novel human atypical SLC transporters, MFSD4A and MFSD9, and their neuroanatomical distribution in mice

**DOI:** 10.1371/journal.pone.0186325

**Published:** 2017-10-19

**Authors:** Emelie Perland, Sofie Victoria Hellsten, Nadine Schweizer, Vasiliki Arapi, Fatemah Rezayee, Mona Bushra, Robert Fredriksson

**Affiliations:** 1 Molecular Neuropharmacology, Department of Pharmaceutical Biosciences, Uppsala University, Uppsala, Sweden; 2 Functional Pharmacology, Department of Neuroscience, Uppsala University, Uppsala, Sweden; University of Queensland Diamantina Institute, AUSTRALIA

## Abstract

Out of the 430 known solute carriers (SLC) in humans, 30% are still orphan transporters regarding structure, distribution or function. Approximately one third of all SLCs belong to the evolutionary conserved and functionally diverse Major Facilitator Superfamily (MFS). Here, we studied the orphan proteins, MFSD4A and MFSD9, which are atypical SLCs of MFS type. Hidden Markov Models were used to identify orthologues in several vertebrates, and human MFSD4A and MFSD9 share high sequence identity with their identified orthologues. MFSD4A and MFSD9 also shared more than 20% sequence identity with other phylogenetically related SLC and MFSD proteins, allowing new family clustering. Homology models displayed 12 transmembrane segments for both proteins, which were predicted to fold into a transporter-shaped structure. Furthermore, we analysed the location of MFSD4A and MFSD9 in adult mouse brain using immunohistochemistry, showing abundant neuronal protein staining. As MFSD4A and MFSD9 are plausible transporters expressed in food regulatory brain areas, we monitored transcriptional changes in several mouse brain areas after 24 hours food-deprivation and eight weeks of high-fat diet, showing that both genes were affected by altered food intake *in vivo*. In conclusion, we propose MFSD4A and MFSD9 to be novel transporters, belonging to disparate SLC families. Both proteins were located to neurons in mouse brain, and their mRNA expression levels were affected by the diet.

## Introduction

Membrane-bound transporter proteins translocate molecules over cellular membranes. Most transporters belong to three major groups [1]; channels move solutes down their electrochemical gradient, primary active transporters use various energy-coupled mechanisms to generate an ion/solute gradient to constitute movements, and secondary active transporters utilise existing energy to translocate molecules [2]. The largest group of transporters in human are the solute carriers (SLCs) [3], which translocate nutrients, waste and drugs via secondary active or facilitated transport [2, 4]. The SLCs comprise 430 members, of which most are divided into 52 families [5]. 28 of the remaining orphan transporters and the members of 16 SLC families (SLC2; 15; 16; 17; 18; 19; SLCO; 22; 29; 33; 37; 40; 43; 45; 46 and 49) belong to the Major facilitator superfamily (MFS) Protein family (Pfam) clan (clan number: CL0015) [5, 6], and together they constitute the largest group of phylogenetically related SLCs [7]. The remaining orphan transporters comprise the 18 Major facilitator superfamily domain containing (MFSD) proteins (MFSD1, 2A, 2B, 3, 4A, 4B, 5, 6, 6L, 7, 8, 9, 10, 11, 12, 13A, 14A and 14B), the three synaptic vesicles glycoprotein 2 proteins (SV2A, SV2B and SV2C), two SV2 related proteins (SVOP and SVOPL), three sphingolipid transporters (SPNS1-3) and two Unc-93 (UNC93A and UNC93B1) proteins [5, 7–10]. MFSD4A and MFSD9 were the targets investigated in this article. The MFS protein family is proposed to have evolved from a common ancestor [11], but is functionally very diverse [12]. Conserved MFS proteins can be found in all three domains, with protein expression in, for example, bacteria, nematodes, arthropods and chordates [8, 9, 11, 12]. Structurally, MFS transporters are composed of one single-polypeptide [12], which probably arose by duplication of 6-transmembrane segments (TMS), resulting in a 12 TMS protein [11] that folds into a cylinder-shaped transporter [13].

Regarding *Slc* genes, approximately half are expressed in mouse brain areas are involved in food intake and energy production [14]. MFSD proteins are also found in the mouse brain, with expression in neurons [9, 15, 16] and the CNS vascular system [17], but not in astrocytes. Looking at subcellular expression in various rodent and human cell types, MFSD proteins have been detected in both plasma [16, 18] and intracellular [15, 19–22] membranes. It has also been reported that the same protein is expressed both in mouse neuronal plasma membrane [16] and lysosomal membranes in HeLa and rat liver cells [22, 23]. However, it is unclear if this difference in subcellular location is due to cell type divergence, function or evolutionary speciation. Some MFSD proteins have confirmed mRNA expression, where *Mfsd9* levels are detected in both central and peripheral rat organs [8], whereas the *Mfsd4a* expression was uncharacterised. Furthermore, transcription levels of MFSD genes can change after food deprivation and high-fat diet in mice [9, 15, 16, 19, 24] and in mouse hypothalamic N25/2 immortal cells after complete amino acid starvation [25].

The studies of orphan transporters is important, and in 2015 there was a “call for systematic research on solute carriers” [26]. The reason for this request is that SLCs are essential as they control processes like nutrient uptake, ion transport and waste removal [2] and disturbances in membrane transport is associated with diseases [4, 27–29]. Of the studied SLCs, one quarter are linked to diseases in humans [26, 27], and to elucidate location and function of the still orphan transporters could aid in understanding why we get sick. Furthermore, SLCs are highly interesting in pharmacology as they can both mediate drug transport to a certain location and be utilises directly as a drug target [26, 27, 30]. Despite their evidential relevance, only few proteins currently serves as drug targets, and those who are, are used substantially [30]. The reason for this is presumably the little information available of the SLC family, compared to other membrane bound protein families [26]. It has also been proven difficult to express and crystalize these proteins as they are embedded in lipid bilayers [4], and functional characterizations of SLC proteins still relies upon computational structural predictions.

By using Hidden Markov Models, orthologues for human MFSD4A (MFSD4; HGNC number: 25433) and MFSD9 (HGNC number: 28158) were identified in several vertebrates, showing evolutionary conservation. Tertiary protein structures were predicted, suggesting them to be novel plausible transporters, composed of 12 TMS each. With immunohistochemistry, the neuronal expression was mapped in mouse brain, showing staining in areas involved in controlling food behaviours. Moreover, the expression levels of *Mfsd4a* and *Mfsd9* were altered in mouse brain areas in response to food deprivation and high-fat diet.

## Material and methods

### Ethical considerations and animals

The study was approved by, and carried out in accordance with the recommendations of, the local ethical committee in Uppsala (Uppsala Djurförsöksetiska Nämnd, Uppsala district court, under the permit numbers C39/16 and C419/12). Adult C57Bl6/J mice (Taconic M&B, Denmark) were housed in accordance with the Swedish regulation guidelines (Animal Welfare Act SFS 1998:56) and European Union legislation (Convention ETS123 and Directive 2010/63/EU). The animals were euthanized during the light period by either cervical dislocation or transcardiac perfusion of anaesthetised animals.

### Phylogenetic clustering of MFSD4 and MFSD9

Hidden Markov Models (HMM) were calculated for mammalian MFSD4A and MFSD9 protein sequences, using HMMbuild from the HMMER package [31]. The models were used to search the protein datasets obtained from Ensembl version 86 [32] listed in Table 1, to identify related proteins.

**Table 1:** Description of the protein data sets used for the phylogenetic analysis.

Species	Common name	Data set version
*A*. *carolinensis*	Lizards	AnoCar2.0.pep.all
*S*. *cerevisiae*	Yeast	R64-1- 1.pep.all
*C*. *elegans*	Roundworm	WBcel235.pep.all
*D*. *rerio*	Zebrafish	GRCz10.pep.all
*D*. *melanogaster*	Fruit fly	BDGP6.pep.all
*G*. *gallus*	Chicken	Galgal4.pep.all
*G*. *aculeatus*	Three-spined stickleback	BROADS1.pep.all
*Homo sapiens*	Human	GRCh38.pep.all
*M*. *musculus*	Mouse	GRCm38.pep.all

All genomes were obtained from Ensemble version 86.

Sequences were manually curated, and proteins originating from the same locus and pseudogenes were removed. The longest protein from each species was combined in a multiple PSI/TM tcoffee sequence alignment [33]. Subsequently, their relationship was inferred according to the Bayesian approach, as implemented in mrBayes 3.2.2 [34, 35]. This was done to confirm the relations between the identified proteins. The analysis was run via the Beagle library (Ayres et al. 2012), on six chains (five heated and one cold), with two runs in parallel (n runs = 2), for a maximum of 2,000,000 generations.

Sequence similarities between orthologue proteins were calculated using global pairwise alignments, based on the Needleman-Wunsch algorithm [36]. The alignments were built on the annotated proteins listed in Table 2.

**Table 2:** Annotation of MFSD4A and MFSD9 orthologues.

Species	MFSD4A		MFSD4B		MFSD9	
	Annotated name	Accession number Ensembl v.86	Annotated name	Accession number Ensembl v.86	Annotated name	Accession number Ensembl v.86
*A*.*carolinensis*	MFSD4A	ENSACAP00000014002	MFSD4B	ENSACAP00000011183	MFSD9	ENSACAP00000007127
*D*. *rerio*	Mfsd4a	ENSDARP00000032062	MFSD4B	ENSDARP00000101496	Mfsd9	ENSDART00000114323
*G*. *gallus*	MFSD4A	ENSGALP00000000981	MFSD4B	ENSGALP00000024202	MFSD9	ENSGALT00000027111
*G*. *aculeatus*	Mfsd4a	ENSGACP00000014503	Mfsd4b	ENSGACP00000008587	Mfsd9	ENSGACP00000003881
*H*. *sapiens*	MFSD4A	ENSP00000356115	MFSD4B	ENSP00000357840	MFSD9	ENSP00000258436
*M*. *musculus*	Mfsd4a	ENSMUSP00000116282	Mfsd4b	ENSMUSP00000040384	Mfsd9	ENSMUST00000039672

Proteins are clustered into SLC families based on homology, function, structure [37] and sequence identity [2]. MFSD4A cluster phylogenetically with MFSD4B and the SLC29 family, whereas MFSD9 is closest related to the SLC46 family, MFSD10, MFSD14A and MFSD14B [5]. Global alignments were performed according to the Needle approach [36] to calculate sequence identities as a way to study if MFSD4A and MFSD9 could belong to existing SLC families.

### Predicted protein structures of human MFSD4A and MFSD9

Transmembrane segments (TMS) in the human MFSD4A and MFSD9 proteins were predicted using the three topology tools TMHMM server (v. 2.0) [38], Phobius prediction [39, 40] and Sousi [41]. These topology tools incorporate parameters such as hydrophobicity, charge bias, helix lengths and signal peptide predictions [38, 39] when making predictions. The tertiary structures were built using Swiss-Model, a fully automated homology program [42]. A structurally known MFS lactose permease from *E*. *coli* [43] (PDB ID code: 2v8n) was used as template for the MFSD4A model, and the YajR [44] (PDB ID code: 3WDO) MFS protein, found many gram-negative bacteria, was the templet used for the MFSD9 model. The alignments between the proteins of interest and their templates were manually inspected to verify that conserved MFS motifs, like the characteristic cytoplasmic loop between TMS 6 and 7 [45] were matched. Images of the tertiary structures were finalized using Swiss-Pdb Viewer [46] and coloured using Adobe Photoshop CS6.

### mRNA extraction and reverse transcription

Adult male C57Bl6/J mice were sacrificed by cervical dislocation, and the following organs were dissected freshly, as described in [9]: brainstem, cerebellum, cortex, eye, heart, hippocampus, hypothalamus, intestine, kidney, liver, lungs, olfactory bulb, ovary, spinal cord, spleen, striatum, thalamus and thymus (male mice), and uterus (female mice). Blood was collected from male mice via cardiac puncture, mixed with EDTA (1.5mg/ml blood, VWR), and centrifuged to retrieve a pellet for RNA extraction. N = 5 per organ. The samples were mechanically homogenized in a bullet blender (Averill Park, USA) and RNA was extracted using Absolutely RNA Miniprep Kit (Agilent Technologies), following the manufacturer’s instructions. The final concentrations were measured in a spectrophotometer (ND-1000, NanoDrop Technologies). Reverse transcription was performed using the Applied Biosystems High-Capacity RNA-to-cDNA kit (Invitrogen), following the manufacturer’s manuals. 2μg RNA was used as template for each cDNA synthesis. RNA was extracted separately for each organ and individual. The cDNA from each organ was then pooled and diluted to 5ng/μl RNA in sterile water.

### Primer design, quantitative-time PCR and data analysis

Primers for mouse *Mfsd4a* and *Mfsd9* and reference genes (marked *) were designed in Beacon Design 8 (Premier Biosoft, Palo Alto), and listed in Table 3. qPCR mastermix contained 2x DreamTaq Buffer (Thermo scientific), 0.2μl 20mM dNTP, 0.05μl forward and reverse primer (100pmol/μl), 0.5μl of SYBR Green (1:50000; Invitrogen) in TE buffer (pH 7.8), 1μl Dimethyl sulfoxide (Sigma Aldrich) and 0.08μl DreamTaq polymerase (5U/μl, Thermo scientific). 5μl pooled cDNA per reaction was used as template. The measurements were run on iCycler real-time detection instrument (Bio-Rad Laboratories) according to following parameters: 30 sec at 95°C initial denaturation, followed by 50 cycles of 10 sec at 95°C, 30 sec at 55–61°C (optimal temperature for each primer pair) and 30 sec at 72°C, followed by a melting curve (+0.5°C per cycle, 81 cycles at 10 sec intervals, starting from 55°C). Each sample was run in triplicates. Negative controls were included on each plate. All experiments were repeated twice.

**Table 3:** Primer sequences used in the quantitative real-time PCR analyses.

Gene	Forward	Reverse
*Mfsd4a*	5’-gcaaggcttctggcatca-3’	5’-gtaacaggacatttgttcctcct-3’
*Mfsd9*	5’-tggtgtcttgttcagagt-3’	5’-tgtgtaagcaaatctccta-3’
Gapdh*	5’-gccttccgtgttcctacc-3’	5’-gcctgcttcaccaccttc-3’
bTub*	5’-agtgctcctcttctacag-3’	5’-tatctccgtggtaagtgc-3’
Rpl19*	5’-aatcgccaatgccaactc-3’	5’-ggaatggacagtcacagg-3’
H3a*	5’-ccttgtgggtctgtttga-3’	5’-cagttggatgtccttggg-3’
Cyclo*	5’-tttgggaaggtgaaagaagg-3’	5’-acagaaggaatggtttgatgg-3’
Actb*	5’-ccttcttgggtatggaatcctgtg-3’	5’-cagcactgtgttggcatagagg-3’

Raw-data was collected from the MyIQ (Bio-Rad Laboratories) software. Primer efficiencies were calculated using LinRegPCR software and Grubbs test (GraphPad software) was performed to remove outliers. The GeNorm protocol [47] was used to detect stable reference genes, and their geometric mean was used to normalize the data. *Gapdh*, *bTub*, *Rpl19*, *Cyclo* and *Actb* were stably detected between samples, and subsequently used for the normalization. The sample with the highest gene expression for each transporter was set to 100%, and the relative expression level of each tissue was plotted (±SD) in the GraphPad Prism 5 software.

### Western blot to study antibody binding

Antibody binding was verified by western blot on fractionated mouse brain tissue as previously described [9, 15]. Protein amount was 100μg per well and the protein transfer was performed with the Trans-Blot® Turbo™ Mini PVDF Transfer Packs and Trans-blot Turbo Transfer system (Bio-Rad), following the manufacturer’s instructions. Anti-MFSD4A (1:100, rabbit, AV53395, Sigma-Aldrich) and anti-MFSD9 (1:50, goat, sc-247973, Santa Cruz) were used as antibodies, and a molecular weight marker (PageRuler Prestained, Thermo Fisher Scientific) was included as reference on each blot. HRP-coupled secondary antibodies (anti-rabbit and anti-goat (Invitrogen) dilution 1:10000) were added followed by chemiluminescent development using Clarity Western ECL Substrate (Bio-Rad). Staining was visualized using a CCD camera (Bio-Rad). Glycosylation sites for the mouse proteins were predicted using the NetOGlyc 4.0 Server from CBS Predictions Servers [48].

### Preparation of mouse brain sections

See [9] for precise procedures regarding fixation, paraffin embedding and sectioning. In brief, adult male mice C57Bl6/J were anesthetized by i.p. injection of 0.01mg/g body weight sodium Pentobarbital (Apoteket Farmaci, Sweden). The tissue was fixed by transcardiac perfusion with 4% formaldehyde (Histolab, Sweden). Brains were stored in 4% formaldehyde over night before sectioning. For DAB staining, the fixed brains were mounted in 4% agarose (VWR) and cut into 70μm coronal sections using a Leica VT 1200 S vibratome (Leica Microsystems). For fluorescent immunohistochemistry the tissue was embedded in paraffin [9] and cut into 7μm coronal sections using a HM355S microtome (Thermo Scientific).

### Immunohistochemistry staining on paraffin embedded mouse brain sections

Fluorescent immunohistochemistry was performed on 7 μm paraffin embedded coronal sections. The sections were rehydrated before antigen retrieval through 10 min boiling in 0.01mM citric acid (Sigma-Aldrich), pH 6.0. The sections were washed in PBS, before addition of primary antibodies, diluted in 5% milk blocking solution (Blotting grade blocker, Bio-Rad). Anti-MFSD4A (1:50) and anti-MFSD9 (1:50) were co-stained with anti-NeuN (1:400, mouse, Millipore, MAB377) and anti-GFAP (1:400, mouse, Millipore MAB360). Subsequently, the sections washed in PBS before incubated with the secondary antibodies Alexa 488 goat-anti-rabbit, Alexa 488 donkey-anti-mouse, Alexa 594 donkey-anti-mouse and Alexa 594 donkey-anti-goat (Invitrogen), diluted 1:800. The sections were mounted in Mowiol anti-fade mounting medium before imaged in an Olympus fluorescence microscope BX53, with an Olympus DP73 camera. The micrographs were acquired by cellSens Dimension software and show representative staining.

### Colorimetric staining on free floating coronal brain sections

3, 3 –diaminobenzidine (DAB) free-floating immunohistochemistry was performed on 70μm thick coronal brain sections, as previously described in [16], with addition of a 40min incubation in 70°C, a 0.01M citric acid (Sigma-Aldrich) (pH 6.0) step for antigen retrieval, and 4x8 min TBS washes prior to blocking of endogenous peroxidases (10 min incubation in TBS with 10% methanol (Sigma-Aldrich) and 3% H_2_O_2_ (Merck)). Sections were incubated in 1% blocking reagent (Roche Diagnostics) for 1h prior antibody incubation. Anti-MFSD4A and anti-MFSD9 antibodies were diluted 1:200. Secondary antibodies (goat-anti-rabbit IgG (H+L), rabbit-anti-goat (H+L), Vector laboratories) were diluted 1:400 in supermix (TBS, 0.25% gelatine, 0.5% Triton X-100). The avidin-biotin complex (ABC kit; Reagent A, Reagent B (Vector Laboratories), was diluted 1:800 in supermix. To develop the staining, the sections were incubated in 0.08% DAB (Sigma-Aldrich), 0.35% NiCl and 0.035% H_2_O_2_. The sections were placed on gelatinized slides (Menzel Gläser) and dehydrated in an ethanol (Solveco) series ranging from 70–100%, ending with Xylene (Sigma-Aldrich), before mounted in DPX (Sigma-Aldrich). In the screen, several Bregma areas were included, and representative staining patterns are shown. The experiment was repeated twice. Micrographs were taken with a Mirax Pannoramic midi scanner (3d Histech) using the Pannoramic Viewer 1.15.4 RTM software (3dHistech). The brightness of all pixels was increased to 75%.

### Mice exposed to altered nutritional intake, followed by mRNA expression measurements

Since both *Mfsd4a* and *Mfsd9* were detected in mouse brain, with expression in brain areas implicated in food intake and its regulation, hypothalamus, pituitary gland, cortex, striatum, thalamus, brainstem and spinal cord were selected for RNA expression analysis. Male mice were divided into three groups receiving three different diets, as described in [9]. 1) Standard chow (control diet), consisting of 5.0% fat, 21.0% protein, and 51.5% carbohydrates (R3, Lantmännen), 2) standard chow *ad libitum*, and starved for 24h prior euthanasia and 3) fed high-fat western diet (HFD), containing 21.0% fat, 17.2% protein, 43.0% carbohydrates (R638, Lantmännen) for eight weeks to induce obesity, all according to [9]. No deviant behaviours in the mice were observed. At dissection day, the obese mice had a 38.0% ± 9.0% mean weight gain compared to a 12.0% ± 2.3% increase in controls. N = 4 per diet for each brain area. All animals had access to water *ad libitum*. mRNA extraction, cDNA synthesis and qPCR were done as described above.

For the analysis, the geometric means of the three most stable reference genes (*Gapdh*, *H3a* and *Actb*) according to GeNorm calculations were used for normalization of the ct-values±SD. The values from control samples for each area were set to 1, and the test group sample values were stated as relative values. Differences in gene expression between groups were analysed by unpaired t-tests, followed by Bonferroni correction for multiple testing (Graphpad Prism 5). Significance levels were set to *p≤0.0493, **p≤0.00998, ***p≤0.001.

## Results

### MFSD4A and MFSD9 have orthologues in several vertebrates

**Fig 1:**
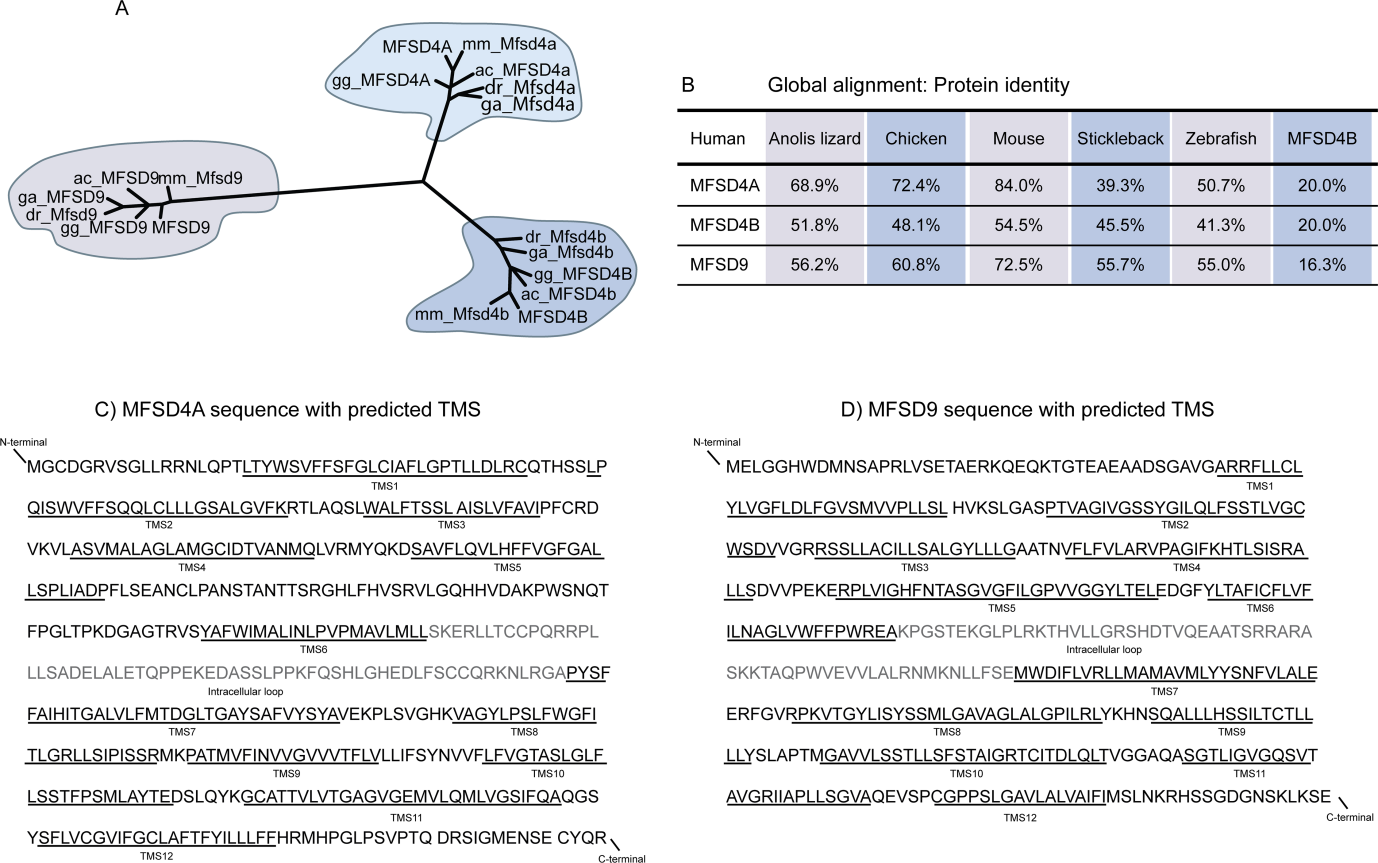
Orthologue clustering and sequence topology. Hidden Markov Models were utilised on proteomes from various species to identify orthologues to human MFSD4A and MFSD9. (A) Schematic representation of the branching order for orthologue proteins. Abbreviations: ac, anolis lizard; dr, zebrafish; ga, stickleback; gg, chicken; mm, mouse. Neither of the proteins was found in yeast, roundworm or fruit fly. MFSD4B was identified as orthologue to MFSD4A. Global pairwise alignments were run to calculate protein identities between human and animal orthologues, as listed in (B). The human protein sequence for MFSD4A (C) and MFSD9 (D) were depicted, where the 12 transmembrane segments (TMS) were underlined (TMS1-12). The first 6 TMS constitute the N-domain which is connected to the C-domain (TMS7-12) via a long cytoplasmic loop (grey). Both the N and C terminals were predicted to be localised on the cytoplasmic side of the membrane.

Several proteomes (yeast, roundworm, fruit fly, zebrafish, three-spined stickleback, lizard, chicken, mouse and human) were scanned with HMMs. The identified proteins were combined in a phylogenetic tree to identify orthologue proteins to human MFSD4A and MFSD9. Both proteins were conserved in all vertebrate data sets studied (Fig 1A). The hits obtained from the yeast, roundworms and fruit fly proteomes did not clustered in proximity with the human proteins. They were discarded as orthologues, even if they still were considered to be related proteins. MFSD4B (synonymous names: KIAA1919 and NAGLT1) was identified as a related protein in all species in the MFSD4A searches. Globally, protein sequence identities were high between the orthologues (Fig 1B), suggesting similar protein function among species. Amongst the organisms examined, human and mouse shared the highest sequence identity, with 84.0% for MFSD4A, and 72.5% for MFSD9. When comparing human MFSD4A with MFSD4B, sequence identity amounted to 20.0% (Fig 1B).

### Shared sequence identity with phylogenetically related MFSD and SLC proteins

Proteins within each SLC family are phylogenetically related [5] and share at least 20% sequence identity to at least one other family member [2]. The shared sequence identity between MFSD4A, MFSD9 and their phylogenetically closest proteins were analysed by global alignments. MFSD4A phylogenetically cluster to MFSD4B and SLC29, while MFSD9 clusters closest to SLC46, MFSD10, MFSD14A and MFSD14B [5]. Analyses of protein sequence identities showed that MFSD4A shared 20.0% sequence identity with MFSD4B; 15.2% sequence identity with SLC29A1; 16.8% with SLC29A2; 14.9% with SLC29A3 and 17.0% with SLC29A4. MFSD9 had 20.6% sequence identity with SLC46A1; 17.2% with SLC46A2 and 10.6% with SLC46A3. Furthermore, MFSD9 was 21.6% identical to MFSD10; 19.1% to MFSD14A and 21.3% to MFSD14B.

### Predicted protein structures for human MFSD4A and MFSD9

The number and structure of possible transmembrane segments (TMS) were assessed based on primary amino acid sequence using TMHMM [38], Phobius [39, 40] and Sousi [41]. All three tools provided similar results concerning the amino acids spanning the membrane. For MFSD4A, all three programs predicted 12 TMS, in accordance with most MFS proteins [11, 13], with N and C terminals on the inside. TMHMM predicted MFSD9 to have 10 TMS, where only eight met the requirement for highest probability, whereas Phobius and Sousi predicted MFSD9 to have 10 TMS.

**Fig 2:**
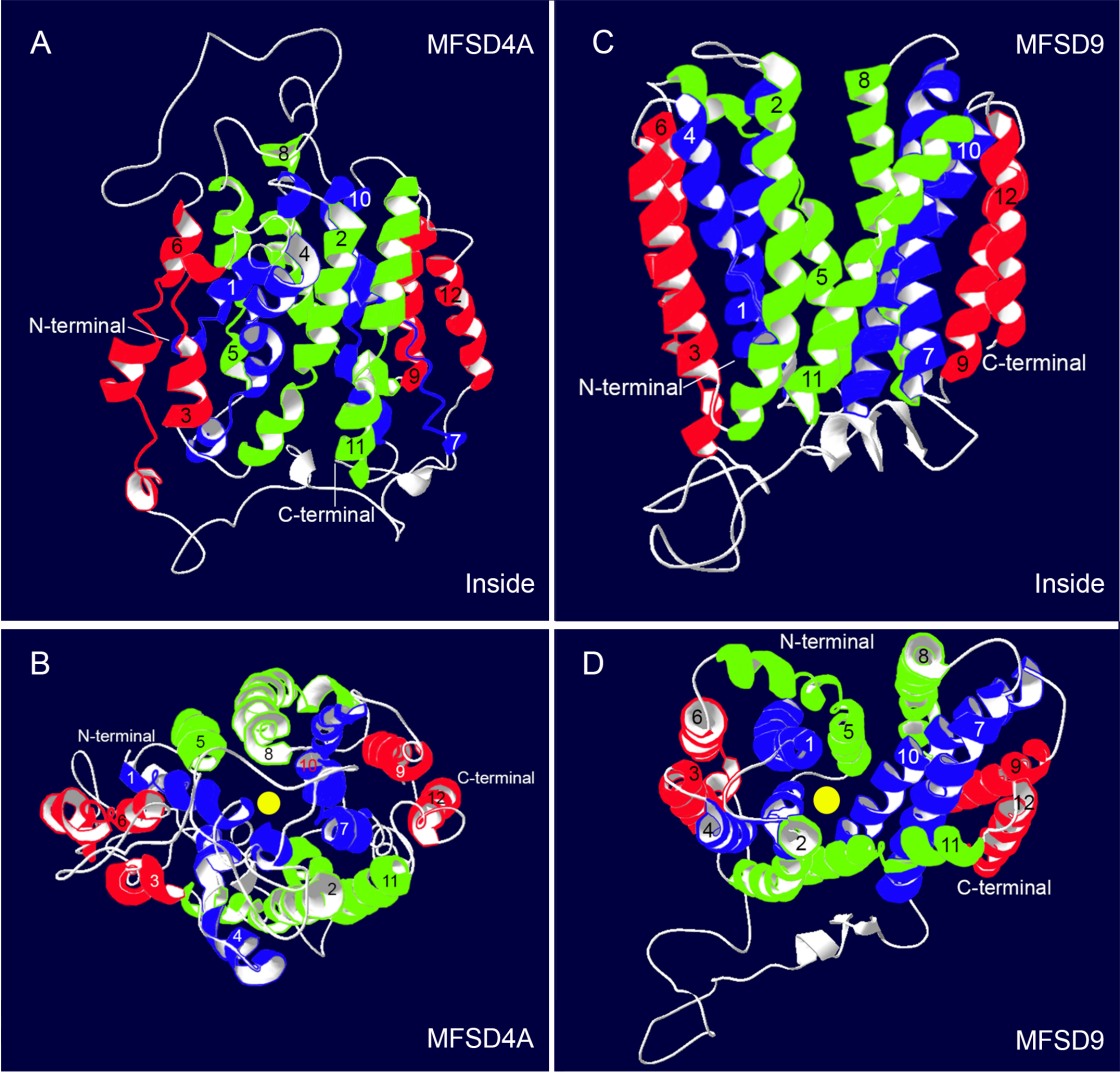
Homology models of MFSD4A and MFSD9. Homology models were built using the SWISS-MODEL [42], with MFS proteins as templates. Both MFSD4A (A, B) and MFSD9 (C, D) were predicted to have 12 transmembrane segments (TMS); where TMS 1, 4, 7 and 10 were depicted in blue, TMS 2, 4, 8 and 11 in green and TMS 3, 6, 9 and 12 in red. In the side view of MFSD4A (A) the N and C terminals are marked. From the top view (B), a potential substrate pore is visible (marked with yellow circle). The same set-up applies for the side view of MFSD9 (C) regarding colours and the N and C terminals. In the top view (D), a possible substrate pore was detected.

Homology models were built using the SWISS-MODEL program [42], in which a structurally known MFS transporter was used as template for each model. Global model quality estimation indicates the reliability of models on a scale range from 0–1, 1 representing total reliability. The MFSD4A model reached a quality score value of 0.40. MFSD9 reached a model quality score of 0.47. Fig 1C and 1D depicts a schematic representation of the proteins topology, where the TMS are detailed according to the homology models. The three dimensional model for MFSD4A resulted in 12 TMS (Fig 2A and 2B), which correlate well with the TMS identified in the secondary structure prediction. Likewise, for MFSD9 12 TMS were identified (Fig 2C and 2D), unlike the 10 TMS previously predicted based on primary amino acid sequence. However, after comparing the TMS identified by the three topology tools with the homology model for the MFSD9 models, TMS3 and 8 from the secondary models corresponded to four TMS in the homology models, providing a final 12 TMS structures, with N and C terminals on the cytoplasmic side of the membrane. For MFSD4A, all except TMS 9 and 12 were incomplete helices, with disrupted intermediate sections, while for MFSD9, TMS 4, 5, 6 and 11 were predicted to be incomplete helices. In both models, TMS 1, 4, 7 and 10 appear to be closest the substrate pore, and they were predicted to contain a long cytoplasmic loop between TMS 6 and 7 (Fig 2A and 2C), two traits common for MFS protein structures [45]. Both peptides folded into a cylinder, through which molecules possibly could be transferred (Fig 2B and 2D).

### *Mfsd4* and *Mfsd9* mRNA expression in both central and peripheral regions

**Fig 3:**
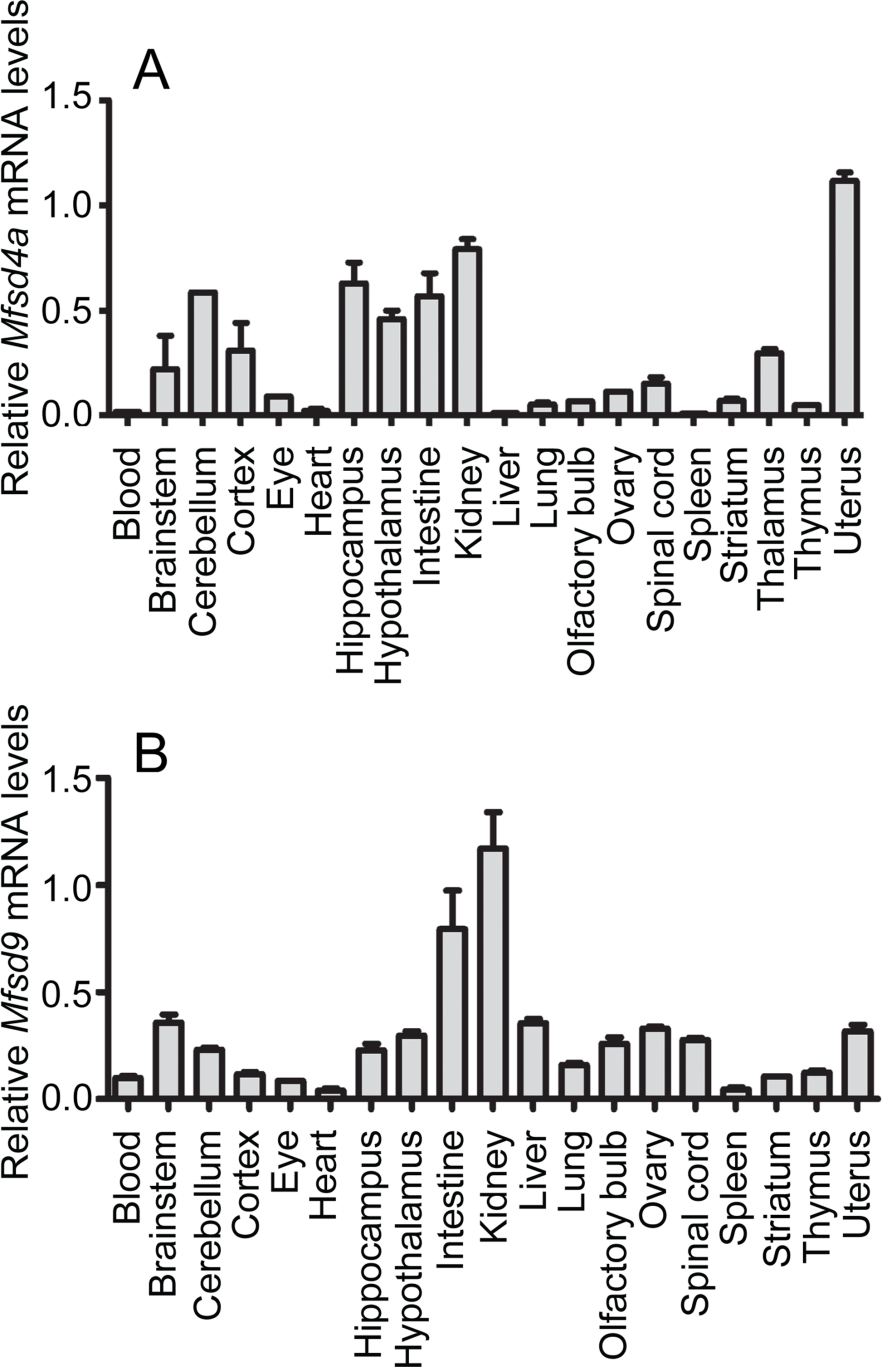
mRNA expression in wildtype and food controlled mice. Relative mRNA expression of *Mfsd4a* and *Mfsd9*, in central and peripheral tissues from adult C57Bl6/J mice, was analysed using qPCR. Samples were made from tissue collected from five animals per organ. The mRNA was normalized against the geometric mean of the reference genes *Gapdh*, *bTub*, *Rpl19*, *Cyclo* and *Actb*. The relative expression levels (±SD) were plotted. *Mfsd4a* (A) and *Mfsd9* (B) were detected in both central and peripheral tissues.

To gain a comprehensive understanding of *Mfsd4a* and *Mfsd9* expression in mice, mRNA levels were measured using quantitative real-time PCR (qPCR). Both genes were expressed in the nervous system and in peripheral tissues (Fig 3). In general, *Mfsd4a* had higher relative expression in central than peripheral areas. *Mfsd4a* was relatively high expressed (normalized relative expression ± SD) in cerebellum, hippocampus and hypothalamus, with slightly lower levels in brainstem and cortex (Fig 3A). Variation in mRNA levels between the peripheral organs measured was larger, with highest relative levels in intestines and kidneys, and lowest in the heart, liver and spleen. *Mfsd9* was also detected throughout all organs tested (Fig 3B), where no discrepancy could be detected between CNS and peripheral expression. However, there was variation within CNS and the periphery, where, for example, the brainstem sample expressed higher *Mfsd9* mRNA levels than striatum, and kidney had higher relative *Mfsd9* expression than the eyes. High levels of *Mfsd9* were also detected in intestine and kidney.

### Western blot to study antibody binding

**Fig 4:**
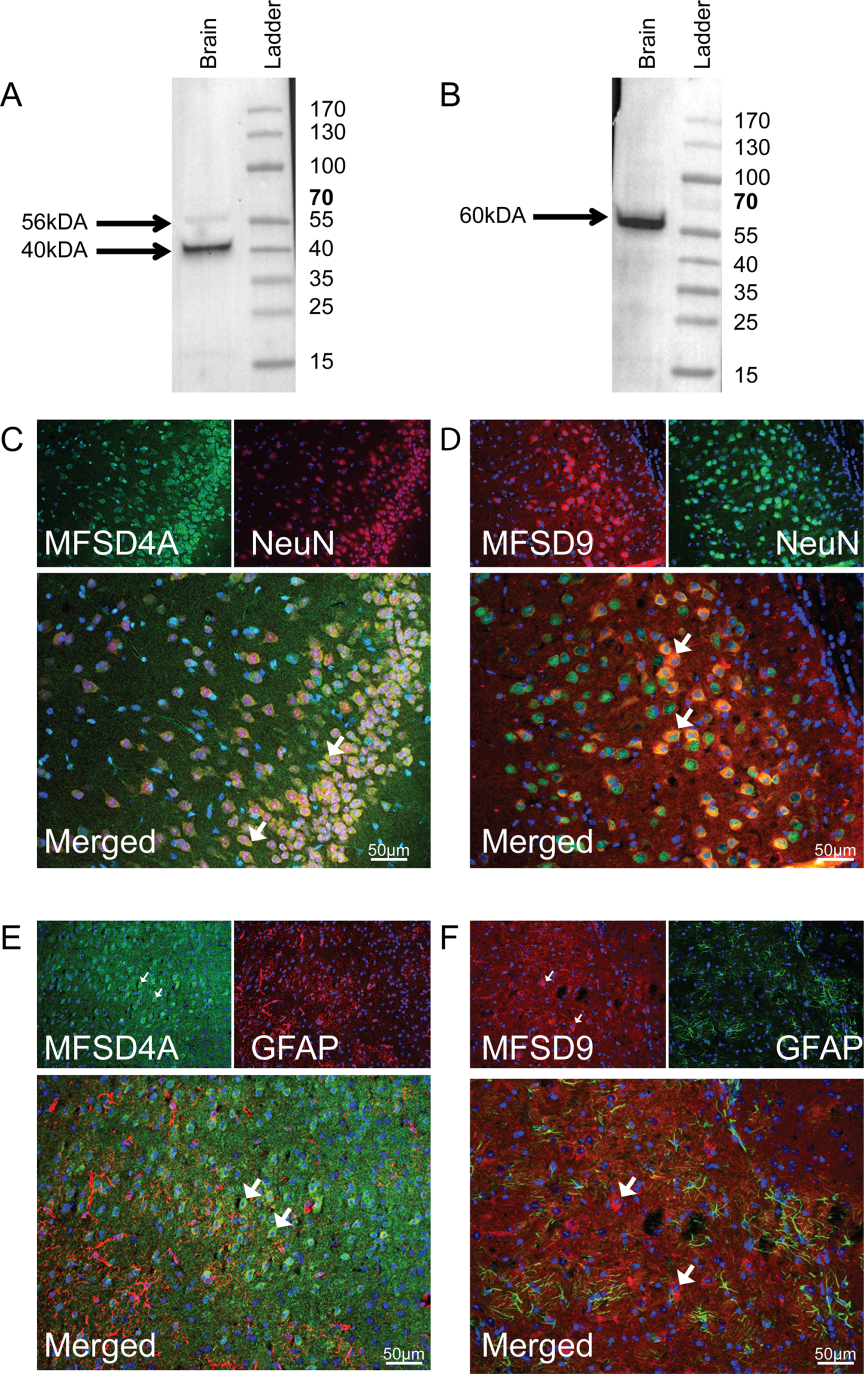
Antibody verification and fluorescent protein staining. Antibodies used in the histological methods were verified using western blot with proteins from a fractionated mouse brain. (A) Staining for MFSD4A gave two bands,40 and 56 kDa (expected sizes at 40 and 56 kDa), in the brain sample and (B) staining for MFSD9 showed a band at 60 kDa (expected size at 51 kDa) in the mouse brain. 7μm coronal adult mouse brain sections were stained for MFSD4A and MFSD9, together with the neuronal marker NeuN and astrocytic marker GFAP, and representative staining is shown. Both MFSD4A (green in C) and MFSD9 (red in D) staining co-localised with NeuN. No overlay was detected between MFSD4A (green in E) and GFAP (red in E), or MFSD9 (red in F) and GFAP (green in F). DAPI was used as a nuclei marker and stained in blue.

Commercially available antibodies were used for protein staining. Antigen binding was confirmed for each antibody by western blot run on fractionated mouse brain. The blot for MFSD4A produced two bands, one at 40 kDA and one at 56 kDA in the mouse brain (Fig 4A), which corresponded with predicted sizes of the isoforms having 56kDA (Ensembl number: ENSMUSP00000125558 and ENSMUSP00000107989) and 40kDA (Ensembl number: ENSMUSP00000039635) as molecular weights. MFSD9 bound at 60kDa (Fig 4B), corresponding to Ensembl number: ENST00000258436.9, having a molecular weight at 51kDA. The band was slightly larger than predicted, likely due to post-translational modifications such as glycosylations. MFSD9 was predicted to contain 12 possible glycosylation sites, as found by analysis via NetOGlyc 4.0 [48]. MFSD4A was predicted to have one possible glycosylation site. The western blot indicated that both antibodies were specific for their respective target.

### Identified MFSD4A and MFSD9 staining in mouse brain neurons

To characterize distribution of both MFSD4A and MFSD9 in mouse brain tissue, fluorescent immunohistochemistry was performed, including the neuronal marker NeuN [49] and the astrocytic marker GFAP [50]. Both MFSD4A and MFSD9 co-localised with NeuN (Fig 4C and 4D), but not with GFAP (Fig 4E and 4F).

### Immunohistochemistry staining of MFSD4A in mouse brain

**Fig 5:**
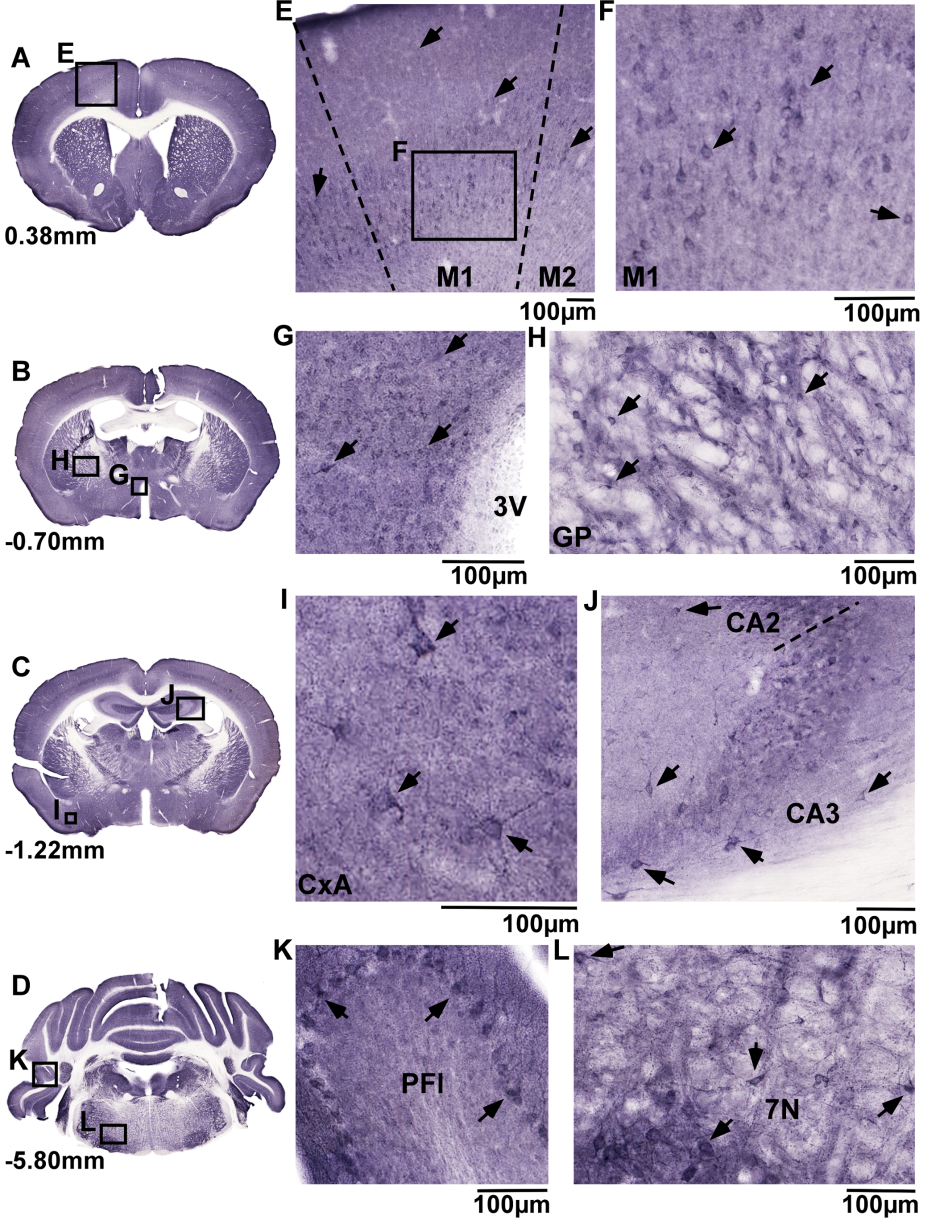
Neuroanatomical distribution of MFSD4A. DAB immunohistochemistry staining on 70μm sections from adult mice brains displaying global MFSD4A protein staining (A-D), with representative close ups (E-L). Cell staining was seen in cortex (E), layer 5, here displayed in primary motor cortex (M1) (F), in hypothalamus along the third ventricle (3v) (G) and globus pallidus (GP) (H). MFSD4A staining was detected along cellular projections, as seen in cells in the cortex-amygdala transition zone (CxA) (I). Stained cells were seen in hippocampal areas CA2 and CA3 (J), the Purkinje cell layer in cerebellum (K) and brainstem nuclei such as the facial nucleus (7N) (L). Bregma regions correspond to [51], and scale bars represent 100μm.

As both proteins were located to neurons, the global neuroanatomical distribution was analysed with DAB immunohistochemistry on 70μm coronal brain sections. Fig 5 displays representative MFSD4A staining, in whole brain sections at bregma 0.38mm; -0.70mm; -1.22mm and -5.80mm (Fig 5A–5D), and selected magnified areas (Fig 5E–5L). Specific layered staining for MFSD4A in cortex layer 5 (Fig 5E), and in the magnified cortex-picture projections were visible (Fig 5F). Labelling of interspersed cells was present in hypothalamic areas around the third ventricle (3V) (Fig 5G), globus pallidus (Fig 5H), amygdala nuclei, starting in the cortex-amygdala transition zone (Fig 5I), and in the CA2 and CA3 region in the hippocampus (Fig 5J). Moreover, distinctly layered staining was observed in the cerebellar cortex, where MFSD4A clearly marked the Purkinje cell layer and their dendritic trees reaching the molecular layer in the paraflocculus (Fig 5K). Staining was seen in the plasma membrane of the soma, as well as in the neuronal projections (Fig 5F and 5I–5K). Finally, dense MFSD4A staining was observed in the facial nuclei of the brain stem (Fig 5L).

### Immunohistochemistry staining of MFSD9 in mouse brain

**Fig 6:**
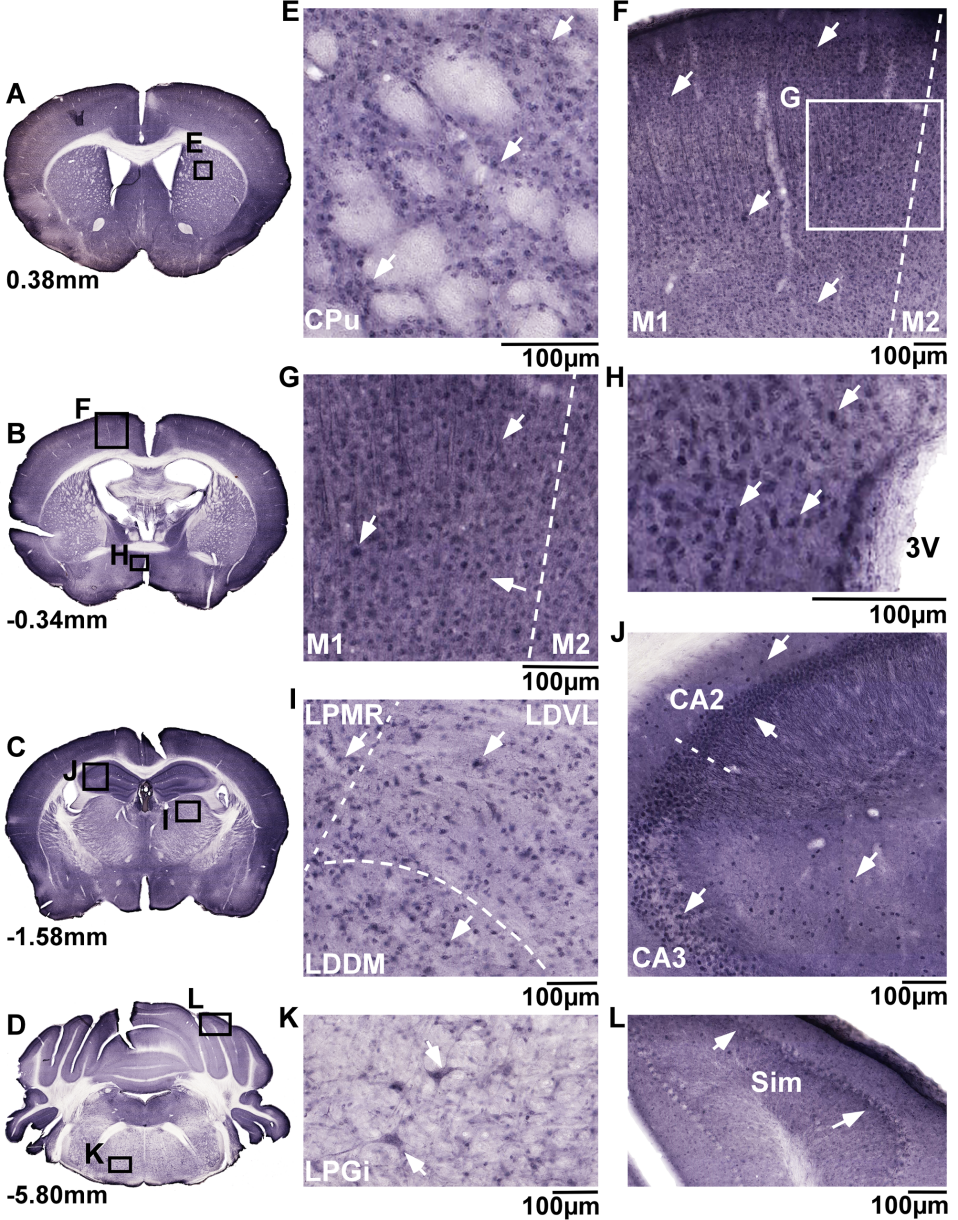
Neuroanatomical distribution of MFSD9. MFSD9 histology was displayed using colorimetric staining on 70μm coronal sections from adult mouse brains. Overview micrographs (A-D), with magnifications (E-L) are showed. MFSD9 staining in striatum (CPu) (E), cortex (F) with possible projections stained in layer 4 and 5 (G) and hypothalamic areas around third ventricle (3V) (H). In thalamus cell bodies were marked by MFSD9 (I). In hippocampus, both soma and projections were detected in CA2, while only soma was seen in CA3 (J). A close up of cells in brainstem (K) and the Purkinje cell layer (L) showed prominent staining. Bregma regions were according to [51], and scale bars represent 100μm.

MFSD9 displayed a more abundant staining pattern from rostral to caudal parts of the mouse brain, as showed at bregma 0.38mm; -0.34mm; -1.58mm and -5.80mm (overview images: Fig 6A–6D; with adjacent magnified micrographs Fig 6E–6L). Densely interspersed staining of MFSD9 was found in the matrix of the striatum (Fig 6E). Evenly distributed staining was visualized throughout cortex (Fig 6F) with stained possible projections in cortex layer 4 and 5 (Fig 6G). MFSD9-positive neurons were found throughout the hypothalamus, around third ventricle (Fig 6H) and in thalamic cells, (Fig 6I). Immunostaining was detected in the fields CA2 and CA3 in hippocampus, where projections from the pyramidal cell layer towards deeper hippocampal layers could be observed in CA2 stretching into the dorsal part of CA3 (Fig 6J). In brainstem, MFSD9 staining was observed in a group of large cells, in which the staining was distributed evenly throughout the soma and projections (Fig 6K). In the cerebellar cortex, the Purkinje cell layer distinctly stained positive for MFSD9 (Fig 6L).

### Expression levels of *Mfsd4a* and *Mfsd9* were affected by altered nutrient intake

**Fig 7:**
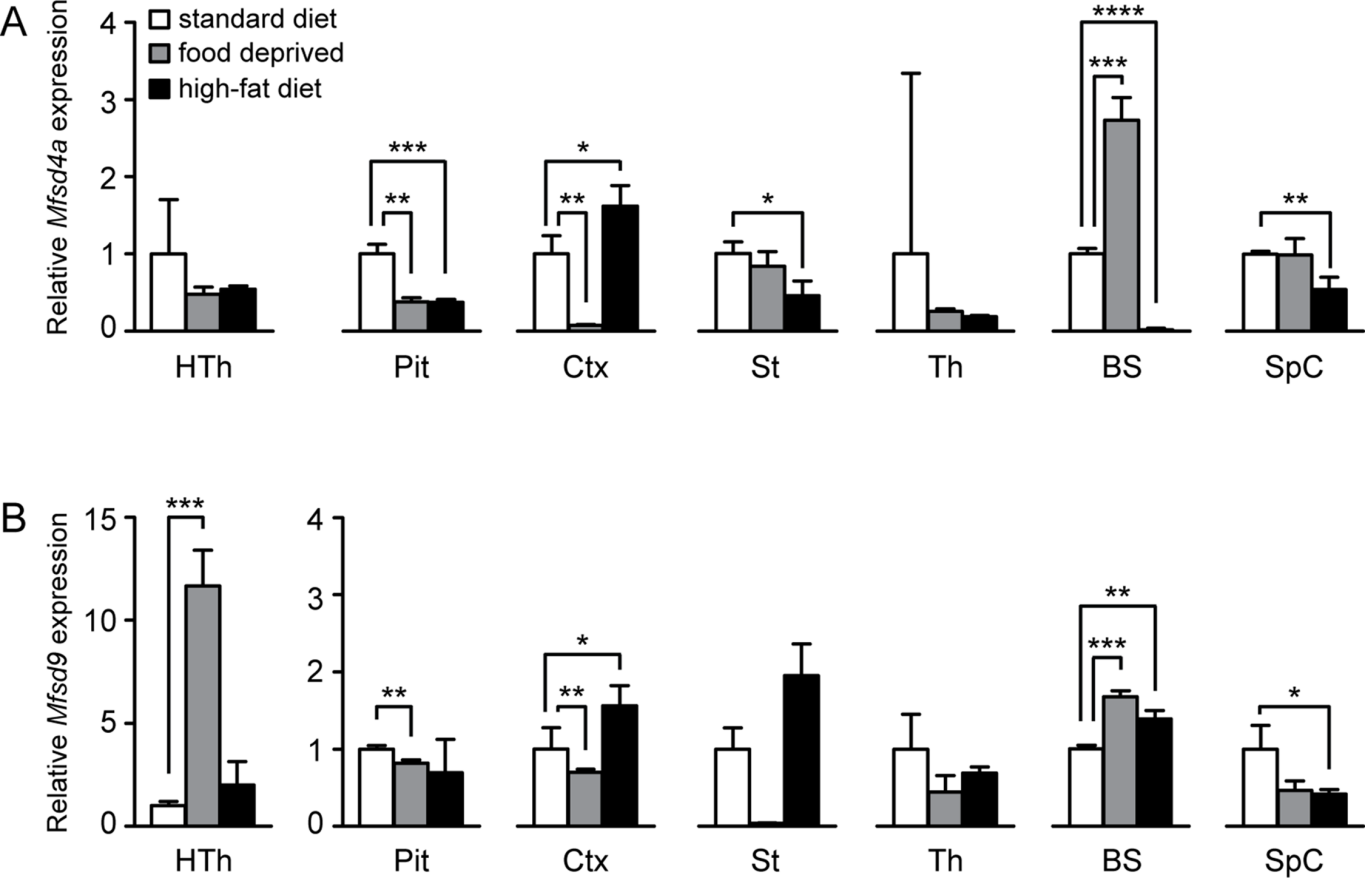
Transcription levels are altered upon changed food intake. To investigate how nutritional status affected transcription levels of *Mfsd4a* and *Mfsd9*, mice were exposed to different food paradigms, 24h food deprivation and eight weeks of high-fat diet, and compared with chow fed mice. N = 4 per area and food paradigm. Relative mRNA levels for *Mfsd4a* (A) and *Mfsd9* (B) are depicted (±SD). Significance levels were set to *p≤0.0493, **p≤0.00998, ***p≤0.001 and ****p<0.0001 after Bonferroni multiple comparison corrections. Abbreviations: HTh, hypothalamus; Pit, pituitary gland; Ctx, cortex; St, striatum; Th, thalamus; BS, brainstem and SpC, spinal cord.

As several MFSD genes are known to respond to altered nutritional status, mice were exposed to three food paradigms, normal chow, food restriction and high-fat diet (HFD). mRNA levels for *Mfsd4a* and *Mfsd9* were measured in the following brain areas: hypothalamus, pituitary gland, cortex, striatum, thalamus, brainstem and spinal cord. For *Mfsd4a* (Fig 7A), no alterations in transcription levels were detected in hypothalamus or thalamus. In pituitary gland, mRNA levels were reduced both by starvation (p = 0.0012) and HFD (p = 0.0010), whereas in cortex, a reduction was seen after food deprivation (p = 0.0023), while the levels were increased by HFD (p = 0.041). Neither the mRNA levels in striatum nor spinal cord were changed due to starvation, whereas HFD down-regulated the *Mfsd4a* expression (Striatum, p = 0.018; Spinal cord, p = 0.0082). Finally, in brainstem, expression levels were up-regulated by starvation (p = 0.0006), and down-regulated in the HFD mice (p<0.00001).

*Mfsd9* was affected by the food paradigms (Fig 7B). Food deprivation increased the expression levels in hypothalamus (p = 0.0005), whereas HFD provided no effects. In pituitary gland, starvation caused a reduction of the expression (p = 0.0082), while HFD had no effects. The cortex responded with down-regulation to starvation (p = 0.0040), while HFD enhanced the *Mfsd9* levels (p = 0.030). No significant changes were seen either in striatum or thalamus. Furthermore, both starvation and HFD up-regulated the *Mfsd9* levels in brainstem (starvation, p = 0.0002; HFD, p = 0.0050), whereas spinal cord was resistant toward food deprivation, but reduced by HFD (p = 0.034). In conclusion, both genes were affected by the nutritional status *in vivo*.

## Discussion

The MFS [52] constitutes the largest superfamily of phylogenetically related secondary active and facilitative transporters [11, 45]. In humans, these types of transporters constitute the solute carrier (SLC) family, of which approximately 30% contain the MFS fold [5]. Among the SLCs of MFS type, there is a subclass of orphan permeases denoted MFSD proteins. We identified MFSD4A and MFSD9 orthologues in several species that could be used when searching the proteins functions, and we predicted the three dimensional structures of human MFSD4A and MFSD9. Moreover, we elucidated the neuroanatomical distribution of the transporters in mice to get a comprehensive understanding of their distribution patterns. Since MFSD4A and MFSD9 are probable transporters involved in nutritional uptake and/or ion transport, we analysed the impact food deprivation and high-fat diet had on their expression levels.

Homologous MFS proteins are recognized in all organismal phyla [11, 52], and the human MFSD proteins have identified orthologues in bacteria, archaea and eukaryotic domains [8, 9, 16, 53]. MFSD4A and MFSD9 were estimated to be comparably young proteins, as relative proteins were only identified in vertebrates, of which orthologues from human and mice shared around 80% of the amino acids. This suggests that both proteins emerged late in evolution, likely to perform specialized tasks in higher species. As SLCs with high sequence similarity usually have similar substrate profiles [54], it is plausible they also share expression patterns. The high sequence identity between the human and mouse proteins suggests that the proteins share function, which makes mice a well-suited model to use when searching for the transporters’ mechanisms. Since orthologues were found in chicken and zebrafish as well, there are more good alternative animal models that could be used when elucidating the proteins functions.

We identified MFSD4A and MFSD4B as related and they shared 20% of the amino acids, meaning they meet the criteria for belonging to the same SLC family [2, 37]. As family members, it is possible that they share a common substrate and mechanism. As MFSD4B is a known sodium dependent sugar transporter [21, 55], it is possible that also MFSD4A transports sugars. MFSD9 shares both phylogenetic branching [5] and more than 20% sequence identity with SLC46A1, MFSD10, and MFSD14B, suggesting they could all be members of the SLC46 family. However, they possess different substrate profiles. SLC46A1 is a folate transporter [56], while MFSD10 (TETRAN) transports organic anions [18].The substrate for MFSD14B is unknown, but due to its high sequence identity (67.7%) with the predicted sugar transporter MFSD14A [57], it is presumed that MFSD14B transports sugar. The substrate for MFSD9 remains to be revealed.

MFSD4A and MFSD9 are similar in sequence and phylogeny with known SLCs, suggesting they are transporters as well. To assess this issue, we used online topology prediction tools to identify possible transmembrane segments (TMS), followed by homology modelling to predict their structures. The topology tools rely on the properties of the amino acid sequence, such as hydrophobicity, charge bias and helix lengths [38, 39] to calculate possible TMS that could span the membrane, whereas the homology models were built in comparison to a structurally determined protein. The optimal set-up for homology modelling would be to use orthologue proteins as templates, but since MFSD4A and MFSD9 lack known related proteins in bacteria, and have no orthologue with determined structure, this cannot be done. Therefore, structurally known MFS proteins were used as templates, and even though the overall sequence identities were low, the conserved MFS domains constitutes good hallmarks when aligning the sequences to the templates [58], and the models were considered reliable.

For MFSD4A, all structural models suggested 12 TMS, and for MFSD9 the prediction tools identified only 10 TMS, while the homology model identified 12 TMS. Under such circumstances the results from the homology modelling holds higher validity, as the protein of interest is aligned against a protein with known structure. As an example, the characteristic long cytoplasmic loop between TMS 6 and 7 was identified in both MFSD4A and MFSD9, and it aligned well with the templates. When comparing the amino acids in the 10 predicted TMS in the secondary models for MFSD9 with the 12 TMS found in the homology model, it was evident that all tools identified the same TMS. However, in the tertiary model, TMS 3 and 8 were divided into four helices. Both models contained incomplete helices, suggesting a lower hydrophobicity index for those helices. This does however not mean that these proteins lack the standard helical configuration. This could be due to hydrophilic residues pointing toward the water filled transport pore or that these hydrophilic residues were shielded from the lipid bilayer by other parts of the protein. As TMS 1, 4, 7 and 10 directly constitute the transport path, and are located in the core of the transporter [45], they can be amphipathic depending on the substrate of the transporter. As these four TMS in the MFSD4A predicted structure mainly consisted of hydrophobic and neutral amino acids, it suggests a non-polar substrate, possible sugars as for MFSD4B [21, 55]. For MFSD9, TMS 1, 4, 7 and 10 contained hydrophobic, hydrophilic and neutral amino acids, suggesting it could translocate charged substrates. Finally, to our knowledge, there is no conclusive evidence supporting a 10 TMS model for MFS proteins, even though it has been investigated [11]. Consequently, we suggest both MFSD4A and MFSD9 to have 12 TMS, composed of the N and C domains.

Commercially available antibodies were used for protein staining, and there are several ways to verify antibodies specificity. A refined way is to use blocking peptides followed by measurements of reduced antibody binding. There are also the possibilities to create knockout mice or knockout cells to show that the antibodies have no cross reactivity. However, for MFSD4A and MFSD9 there were, at present, neither blocking peptides available, nor any confirmed knockout mice or knockout cells to utilise. The specificity of antibodies can also be studied using siRNA knockdown strategies in cultured cells, but both MFSD4A and MFSD9 maintain low protein levels in rodent cell cultures and the proteins cannot be detected. Consequently, we used western blot to ensure the antibodies’ accuracy. With western blot it was verified that both antibodies bound epitopes on proteins having correct predicted sizes, but cross-reactivity cannot be excluded. The histology displays the likely distribution of MFSD4A and MFSD9.

Based on the results presented herein, MFSD4A and MFSD9 are suggested to be novel SLC transporters, belonging to disparate SLC families. That *Mfsd4a* and *Mfsd9* were highly expressed in intestines and kidneys increases the possibility of involvement in nutritional regulation. Since approximately half of all known *Slc* genes present in mouse brain areas are involved in the regulation of food intake and energy production [14], the distribution of MFSD4A and MFSD9 was studied in mouse brain. This showed specific protein staining in food controlling areas such as brainstem [59], hypothalamus [60] and striatum [61], and it corresponded well with measured relative mRNA levels. Since MFSD4A and MFSD9 probably transport nutrients we decided to study if and how they were affected by changed food intake in food regulatory areas. 24 hours of food deprivation was analysed to study acute effects, while 8 weeks of HFD mirror how the genes were affected by food-induced obesity in mice. Diet changes like these can in mice, as in humans, cause the metabolic syndrome [62], with effects like hyperinsulinemia, hyperglycaemia and hypertension. This could contribute to altered mRNA levels and the effects measured could be a resolute of other confounding factors. In general, both genes had similar response to the diets, with some exceptions: in hypothalamus, *Mfsd4a* was unaffected, whereas there was a prominent increase of *Mfsd9* after food deprivation. This up-regulation could imply a required increase in uptake of certain molecules. Due to the phylogenetic and sequential resemblance between MFSD9 and MFSD14A, the *Mfsd9* increase could be a response to the diminished sugar intake, as MFSD14A is a predicted sugar transporter [57]. Both the arcuate nucleus [63, 64] and ventromedial nucleus [64, 65] of the hypothalamus contain specialized glucose-sensitive cells, and perhaps MFSD9 is involved in this regulation. In striatum it was the opposite; *Mfsd4a* was reduced by high-fat diet, while *Mfsd9* remained normal. Such reduction of expression upon exposure to HFD suggests that the system has reached satiety, and that the cells abolish the uptake mechanisms.

In conclusion, that human MFSD4A and MFSD9 proteins were predicted to have the MFS structural appearance, and that they phylogenetically cluster with SLCs suggest they could function as transporters. That protein staining and mRNA expression were detected in food regulatory areas, and that transcriptional changes were measured after altered food intake, suggests involvement in nutrient intake or regulation.
