# Correction: Structural prediction of two novel human atypical SLC transporters, MFSD4A and MFSD9, and their neuroanatomical distribution in mice

**DOI:** 10.1371/journal.pone.0197417

**Published:** 2018-05-10

**Authors:** Emelie Perland, Sofie V. Hellsten, Nadine Schweizer, Vasiliki Arapi, Fatemah Rezayee, Mona Bushra, Robert Fredriksson

The second author's name appears incorrectly. The correct name is: Sofie V. Hellsten.
